# Feasibility and performance of continuous glucose monitoring in hyperglycemia after lung transplantation

**DOI:** 10.3389/frtra.2023.1282215

**Published:** 2024-01-04

**Authors:** Juan M. Munoz Pena, Kimberly Algarra, Hannah Kennedy, Man Chong Leong, Ramzi G. Salloum

**Affiliations:** ^1^ Division of Endocrinology, Diabetes & Metabolism, University of Florida, Gainesville, FL, United States; ^2^ Department of Biostatistics, University of Florida, Gainesville, FL, United States; ^3^ Department of Health Outcomes and Biomedical Informatics, University of Florida, Gainesville, FL, United States

**Keywords:** PTDM, CGM (continuous glucose monitoring), post transplant diabetes mellitus, lung transplant, continuos glucose monitoring, hyperglycemia

## Abstract

**Background:**

Post-Transplant Diabetes Mellitus (PTDM) affects 20%–40% of lung transplant recipients within five years, impacting rejection, infection, cardiovascular events, and mortality. Continuous glucose monitoring (CGM) is used in diabetes but not well-studied in PTDM.

**Objective:**

This study assessed CGM performance in detecting hypoglycemia and hyperglycemia post-lung transplantation, compared to self-monitoring blood glucose.

**Methods:**

A prospective pilot study included 15 lung transplant patients (mean age 58.6 years; 53.3% men; 73.3% with pre-transplantation diabetes) managing hyperglycemia with insulin. Patients used a blinded CGM and self-monitored glucose for ten days. Data were categorized (% time in range, % high, % very high, % low, % very low) and compared using paired t-tests.

**Results:**

CGM showed superior hyperglycemia detection. Mean differences for “% very high”, “% high”, and “% high and % very high” were 7.12 (95% CI, 1.8–12.4), 11.1 (95% CI, 3.5–18.8), and 18.3 (95% CI: 7.37–29.24), respectively. No significant difference was found for “% low and % very low”. All patients reported a positive CGM experience.

**Conclusion:**

CGM use post-lung transplantation seems feasible and offers advantages in detecting hyperglycemia and in optimizing glucose management. Study limitations include a small sample size, requiring larger studies to assess glycemic control, hypoglycemia detection, and transplant outcomes.

## 1 Introduction

Lung transplantation is rapidly evolving and requires a multidisciplinary team approach. According to the 2021 Organ Procurement and Transplantation Network (OPTN) report, the 5-year survival in U.S. adults after lung transplantation is 54.3% (1). The incidence of post-lung transplant diabetes mellitus (PTDM) is estimated to be 10%–40% over five years (2). Specific factors for lung transplantation include immunosuppression, such as glucocorticoids, calcineurin inhibitors, and mTOR inhibitors. Notably, cystic fibrosis confers a high risk as approximately 50% of patients already have diabetes at the time of transplantation, and half the remainder will likely develop diabetes after transplantation (2, 3). Cystic fibrosis is the leading indication for pediatric lung transplant in the U.S (43.3% in 2019) (1). Hyperglycemia is associated with adverse outcomes after solid-organ transplantation, including increased risk of infection, rejection, cardiovascular outcomes, and mortality (2).

Lung transplantation mortality appears to be the highest among solid-organ transplantations who develop PTDM. In a study by Hackman et al. (4), 210 lung transplant recipients were observed for a median of 3 years. All participants underwent oral glucose tolerance tests pre- and serially post-lung transplantation, and significantly more patients with either diabetes mellitus (pre or post-transplant) died or required redo transplantation compared to patients without persistent diabetes (26% vs. 37%).

Current approaches for glucose monitoring consist of traditional self-monitoring of blood glucose (SMBG) using fingerstick testing. Continuous glucose monitors (CGM) are devices placed over the skin that measure the interstitial glucose level in real time or intermittently via a small sensor inserted under the skin and attached to a small transmitter that sends the data wirelessly to a receiver or a smartphone. CGM has improved significantly over time in terms of accuracy, reliability, and ease of use. Several randomized controlled trials in patients with type 1 and type 2 diabetes have shown positive results in terms of reducing A1c levels and/or episodes of hypoglycemia, and most real-world studies have demonstrated A1c improvement and reduction in acute diabetes complications such as episodes of severe hypoglycemia and hospitalization rates (5). The American Diabetes Association recommends offering CGM to pediatric and adult patients with diabetes on multiple daily injections or continuous subcutaneous insulin infusion or “insulin pumps” who can use devices safely either by themselves or with a caregiver (5).

CGM has several potential benefits for individuals who have undergone lung transplantation, especially those who have diabetes or are at risk of developing diabetes after transplant. CGM can help identify glucose abnormalities at an earlier stage, enabling prompt intervention and management of blood sugar levels; optimize insulin dosing and other diabetes medications, leading to improved glycemic control; reduce the risk of complications associated with uncontrolled glucose levels, such as infections, impaired wound healing, and cardiovascular disease; allow individuals to attain greater flexibility and quality of life; and identify trends and patterns allowing for more personalized and effective diabetes management (5).

Whereas continuous glucose monitoring is common in patients with type 1 and type 2 diabetes mellitus, there is a significant knowledge gap in its application to PTDM.

The objectives of this study were (a) to evaluate the performance of CGM in detecting hypoglycemia and hyperglycemia in the early post-lung transplantation period, compared with traditional self-monitoring blood glucose with fingerstick testing; (b) to evaluate the feasibility of implementing CGM in patients after hospitalization for lung transplantation.

## 2 Methods

We conducted a prospective pilot study at UF Health Heart & Vascular Hospital in Gainesville, Florida, involving patients aged 18–75 who underwent lung transplantation. The participants in this study underwent transplantation during their hospital admission and were subjected to insulin management for hyperglycemia upon discharge (Table 1).

**Table 1:** Patients aged 18–75 years who underwent lung transplantation and had hyperglycemia upon discharge.

Number of patients	15
Age, mean	58.6 (SD 17.9)
Male	8
Female	7
Weight, mean	66.5 (SD 21.14)
Preexisting diabetes	11
Duration of hospitalization, mean	56 days (SD 45.40)
Prednisone dose at discharge in mg/day, mean	10 (SD 7.38)
Indications for transplant	
Idiopathic pulmonary fibrosis	4
COPD	4
Interstitial lung disease	3
COVID ARDS	2
Cystic fibrosis	1
Fibrotic lung disease	1

SD, standard deviation; COPD, chronic obstructive pulmonary disease; COVID ARDS, COVID-19-associated acute respiratory distress syndrome.

Patients were included if they were: (a) able to communicate meaningfully with the investigator and provide written informed consent; (b) aged 18–75 years; (c) had received lung transplantation; (d) had a diagnosis of post-transplant diabetes mellitus; (e) received basal-bolus insulin regimen at discharge; (f) HgA1c <10%; (g) had plans to continue care at the University of Florida Health; and (h) willing to comply with study procedures. Patients were excluded if they were: (a) unwilling to participate; (b) planning to receive primary diabetes care at a location outside of UF Health; (c) enrolled in another study with similar outcomes; or (d) had Type 1 Diabetes Mellitus.

Enrolled patients wore a diagnostic (blinded) CGM at discharge, were instructed to self-monitor glucose levels at home with a glucometer, and mail a complete glucose log with the CGM device after ten days of use. All patients received diabetes education from certified diabetes educators while hospitalized, and an endocrinologist optimized insulin dosing. Patients received a carbohydrate consistent diet during hospitalization and were advised to continue it at home. The CGM utilized was the Dexcom G6 Pro (Dexcom, Inc.) and was applied to patients by the endocrinology team caring for the patient upon discharge. The team consisted of an APRN, PA-C, and an M.D. The device was applied over the skin using Dexcom's one-button automatic applicator. Patients were educated on proper care of the device at home, which consisted of avoiding physical manipulation or contact that could remove the device.

Patients wore the device for ten days, removed it on day 11, and mailed it with the glucose logs. The study team and patients remained blinded to the CGM data until it was downloaded. Patients were also instructed to conduct self-glucose monitoring consisting of finger stick checks four times a day (before breakfast, lunch, dinner, and bedtime).

Data were summarized according to the following categories and compared across both tests: % time in range (70–180 mg/dl); % high (181–250 mg/dl); % very high (>250 mg/dl); % low (50–69 mg/dl); % very low (<50 mg/dl).

In the statistical analysis, we used paired *t*-tests to assess the disparity in hyperglycemia detection between CGM use and self-monitored blood glucose.

In addition, a post-study questionnaire was conducted via phone to assess the feasibility of the study. The questionnaire assessed previous knowledge, user experience, and the impact of CGMs on glycemic control (Table 2).

**Table 2:** Post-study questionnaire.

Questions	Participants *N*, %					
	All (*n* = 15)		Mailed the CGM to the study group (*n* = 13)		Received a CGM report from the study group (*n* = 10)	
1. What did you know about continuous glucose monitoring (CGM) technology prior to this study?						
a. Never heard about it	11	73%				
b. Basic knowledge	3	20%				
c. Moderate knowledge	0	0%				
d. Advanced knowledge	1	7%				
2. Did you have any concerns or fears you had about using a CGM device after lung transplantation? If yes, what were your concerns?	0	0%				
a. No, I was looking forward to it	12	80%				
b. Fear of discomfort or pain	3	20%				
c. Concerns about device accuracy	0	0%				
d. Worries about device maintenance and care	0	0%				
3. How did the CGM device impact your daily routine and activities?	0	0%				
a. No impact	11	73%				
b. Slight disruption	3	20%				
c. Moderate changes	0	0%				
d. Significant changes	0	0%				
4. How would you rate your overall satisfaction with using a CGM device?	0	0%				
a. Very dissatisfied	0	0%				
b. Dissatisfied	0	0%				
c. Neutral	2	13%				
d. Satisfied	5	33%				
e. Very satisfied	7	47%				
5. Did you feel that prescribing the CGM device was appropriate for your case?						
a. Yes, completely appropriate	12	80%				
b. Yes, somewhat appropriate	1	7%				
c. Neutral	1	7%				
d. No, somewhat inappropriate	0	0%				
e. No, completely inappropriate	0	0%				
6. Did you have any difficulties mailing the CGM to the study group?						
a. No, it was simple			13	100%		
b. Minor difficulties			0	0%		
c. Moderate difficulties			0	0%		
d. Major difficulties			0	0%		
7. Did the CGM report help you manage your glucose levels more effectively? If yes, how?						
a. Yes, it helped me adjust insulin doses effectively					10	100%
b. Yes, it helped me prevent hypoglycemia					0	0%
c. No, it didn't provide any significant benefit					0	0%
d. No, I did not receive the report					3	30%
8. Did the CGM device improve your awareness of how food, exercise, and other factors impact your glucose levels? If yes, how?						
a. Yes, it educated me about my glucose patterns					10	100%
b. Yes, it provided helpful charts and graphs					7	70%
c. Yes, it helped me understand the impact of food choices					7	70%
d. Yes, it showed me how exercise affects my glucose levels					4	40%
e. No, it didn't improve my awareness significantly					0	0%
f. No, I didn't receive the report					3	30%
9. How satisfied were you with the support provided by the study team during the study period?						
a. Very dissatisfied					1	10%
b. Dissatisfied					0	0%
c. Neutral					1	10%
d. Satisfied					2	20%
e. Very satisfied					10	100%
10. Would you be interested in using a CGM device in the future? Why or why not?						
a. Yes, I found it comfortable and convenient					10	67%
b. Yes, I want to avoid fingersticks for glucose monitoring					13	87%
c. Yes, it can improve my glycemic control					11	73%
d. No, I prefer other methods of glucose monitoring					0	0%
e. No, it didn't provide significant benefits for me					0	0%

## 3 Results

**Figure 1:**
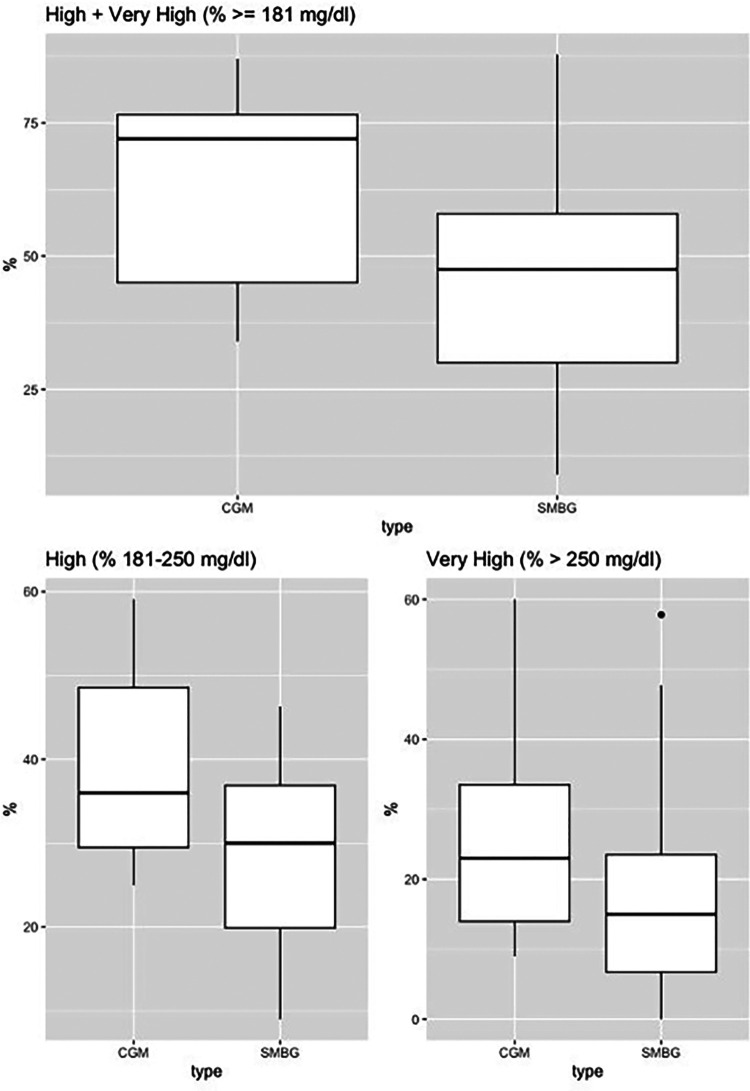
Continuous glucose monitoring vs. self-monitored blood glucose (fingerstick testing). Continuous glucose monitoring vs. self-monitored blood glucose. Boxplot for % high (181–250 mg/dl), % very high (>250 mg/dl), % high + very high (≥181 mg/dl).

The sample included 15 patients (mean age 58.6 years; 53.3% male; 73.3% with pre-transplantation diabetes). There was a significantly higher detection rate for hyperglycemia with CGM use vs. self-monitored blood glucose, as represented in Figure 1. For the “% very high” group, the mean difference was 7.12 [95% confidence interval (CI), 1.82–12.41, *p*-value = 0.012], and for the “% high” group, the mean difference was 11.1 (95% CI, 3.55–18.81, *p*-value = 0.007), and for the combined group, “% high and % very high”, the mean difference was 18.3, (95% CI: 7.37–29.24, *p*-value = 0.002). There was no statistically significant difference for the “% low and % very low” groups.

**Figure 2:**
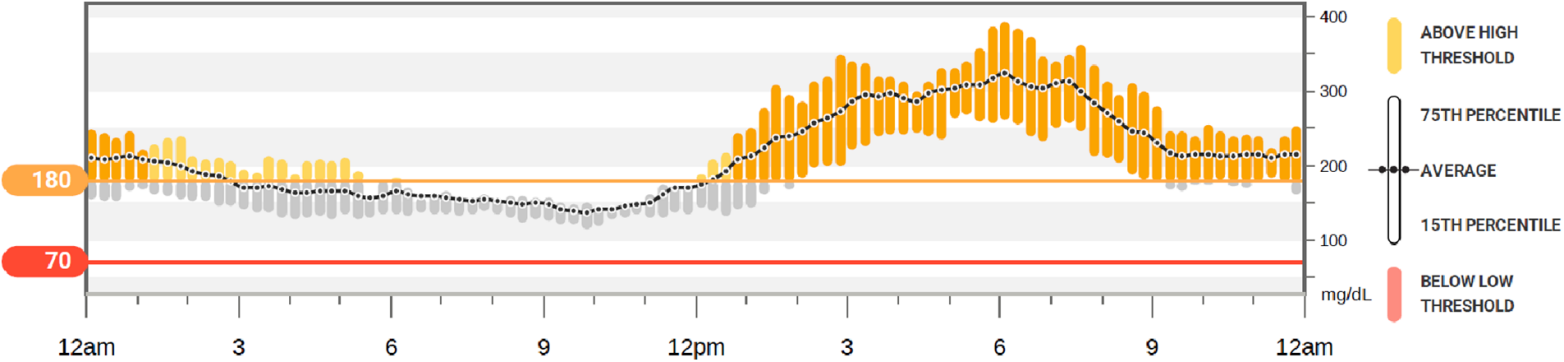
Continuous glucose monitor (sample). Example of the 10-day glycemic control of a participant using glargine and lispro insulin at home after lung transplantation. This demonstrates a pattern of steroid-induced hyperglycemia (fasting euglycemia and post-prandial hyperglycemia) which was commonly found within the participants of this study.

Average CGM metrics: the coefficient of variation was 29%, time in range 40%, high (180–250 mg/dl) 39%, very high (>250 mg/dl) 2%, low (54–70 mg/dl) 0%, and very low (<54 mg/dl) 0% (Figure 2). There was a pattern of post-prandial hyperglycemia which is commonly seen with steroid-induced hyperglycemia (6).

There were no individual factors significantly associated (i.e., *p*-value < 0.05) with higher glucose levels when adjusted for age, gender, weight, duration of hospitalization, prednisone dose at discharge, tacrolimus dose at discharge, indication for transplant, and estimated glomerular filtration rate (eGFR).

### 3.1 Post-study questionnaire results

All patients (*n* = 15) completed the post-study questionnaire. One patient removed the CGM after one day of use due to minor bleeding, one patient did not recall using the CGM, and three patients did not receive the CGM report after mailing it, presumably due to a change in their address.

Among the fifteen patients completing the questionnaire, eleven had never heard of CGMs before the study, twelve had no concerns about using a CGM, and three feared discomfort or pain. Eleven reported no impact on their daily routine and activities, and three reported an impact. All patients reported having a positive experience using CGM and no difficulties mailing it to the study team (Table 2).

Among the ten patients who completed the study and received a CGM report, all reported it helped manage their glucose levels more effectively and improved awareness of their glucose patterns. Seven reported it helped them to understand the impact of food choices, and four, how exercise affects their glucose levels. Finally, all patients were interested in using a CGM in the future.

## 4 Discussion

CGM has revolutionized outpatient glucose monitoring since its US Food and Drug Administration (FDA) approval in 1999. In type 1 and type 2 diabetes mellitus, CGM has been shown to reduce A1c, severe hypoglycemia, and hospitalization rates in several studies and is broadly recommended by the ADA's Standards of Medical Care in Diabetes for outpatient clinical care (5). There are, however, potential factors limiting the accuracy of interstitial glucose monitoring, such as low perfusion index, hypotension, hypothermia, hypoxia, vasopressor use, and edema, as described in early studies in critically ill persons (7).

To our knowledge, no studies utilize this technology in persons with hyperglycemia after discharge from solid-organ transplantation. There is a knowledge gap about the feasibility of CGM use and its accuracy in this population. In addition, little is known about how glycemic control during the early post-transplantation period affects rejection, infection, cardiovascular, and mortality outcomes.

In this real-world study using CGM after discharge from lung transplantation, the feasibility was positive from a patient's and healthcare provider's perspective. This study also showed a significantly higher rate of detection for hyperglycemia with CGM vs. SMBG which could translate into earlier detection of hyperglycemia and improved glycemic control. Of note, there was no documented hypoglycemia in either group.

The hyperglycemia pattern encountered in the study population matches the expected pattern of glucocorticoid-induced hyperglycemia, with normal or near normal fasting BG levels and post-prandial hyperglycemia (6) (Figure 2). Despite optimization of insulin therapy and diabetes education during hospitalization, all enrolled patients experienced post-prandial hyperglycemia. Although the hyperglycemia was mild to moderate, it suggests the need for stronger dietary recommendations, blood glucose monitoring, and optimization of insulin therapy after discharge.

Among the participants, there was an overwhelmingly positive response on CGM usability, ability to improve glycemic control, and overall experience. All participants reported interest in wearing a CGM if given the opportunity.

In summary, CGM use post solid organ transplantation appears to be feasible and may provide significant advantages for the detection of hyperglycemia, monitoring of glucose levels, and management. This study was limited by a small sample size. Larger studies are required to assess outcomes in glycemic control, hypoglycemia detection, and transplant outcomes.

## Data Availability

The raw data supporting the conclusions of this article will be made available by the authors, without undue reservation.
